# Longitudinal single-cell analysis of a patient receiving adoptive cell therapy reveals potential mechanisms of treatment failure

**DOI:** 10.1186/s12943-022-01688-5

**Published:** 2022-12-14

**Authors:** Rihao Qu, Yuval Kluger, Junchen Yang, Jun Zhao, David A. Hafler, Diane S. Krause, Alexey Bersenev, Marcus Bosenberg, Michael Hurwitz, Liliana Lucca, Harriet M. Kluger

**Affiliations:** 1 Department of Pathology, Yale School of Medicine, 333 Cedar St, New Haven, CT 06520 USA; 2 Department of Neurology, Yale School of Medicine, 333 Cedar St, New Haven, CT 06520 USA; 3 Department of Laboratory Medicine, Yale School of Medicine, 333 Cedar St, New Haven, CT 06520 USA; 4 Department of Dermatology, Yale School of Medicine, 333 Cedar St, New Haven, CT 06520 USA; 5 Department of Immunobiology, Yale School of Medicine, 333 Cedar St, New Haven, CT 06520 USA; 6 Department of Medicine, Yale School of Medicine, 333 Cedar St, New Haven, CT 06520 USA

**Keywords:** Adoptive cell therapy, T cell clones, T cell receptor specificity

## Abstract

Adoptive cell therapy (ACT) using tumor infiltrating lymphocytes (TIL) is being studied in multiple tumor types. However, little is known about clonal cell expansion in vitro and persistence of the ACT product in vivo. We performed single-cell RNA and T-Cell Receptor (TCR) sequencing on serial blood and tumor samples from a patient undergoing ACT, who did not respond. We found that clonal expansion varied during preparation of the ACT product, and only one expanded clone was preserved in the ACT product. The TCR of the preserved clone which persisted and remained activated for five months was previously reported as specific for cytomegalovirus and had upregulation of granzyme family genes and genes associated with effector functions (*HLA-DQB1, LAT, HLA-DQA1, and KLRD1).* Clones that contracted during TIL preparation had features of exhaustion and apoptosis***.*** At disease progression, all previously detected clonotypes were detected. New clonotypes appearing in blood or tumor at disease progression were enriched for genes associated with cytotoxicity or stemness (*FGFBP2, GNLY, GZMH, GZMK, IL7R, SELL* and *KLF2*), and these might be harnessed for alternative cellular therapy or cytokine therapy. In-depth single-cell analyses of serial samples from additional ACT-treated patients is warranted, and viral- versus tumor-specificity should be carefully analyzed.

**Supplementary Information:**

The online version contains supplementary material available at 10.1186/s12943-022-01688-5.

## Background

Adoptive cell therapy (ACT) is being studied for multiple tumor types. Initially developed at the National Institutes of Health and subsequently commercialized, it involves tumor resection, ex vivo expansion and activation of T-cells, and non-myeloablative chemotherapy followed by infusion of the ACT product and administration of high-dose interleukin-2. A phase II trial in melanoma is under review for approval by the Food and Drug Administration [1]. TIL therapy exerts its effect by expanding a diverse repertoire of T-cells expressing TCRs that recognize a spectrum of antigens/neoantigens unique to the melanoma. Responses to ACT are reported in up to 50% of patients, with lower response rates in patients previously treated with immune checkpoint inhibitors, but little is known about mechanisms of resistance.

## Case presentation

A man in his thirties was diagnosed with stage IIIC melanoma of the left thigh in March 2016. In August 2017, he developed metastases in the left thigh, left groin and right axilla. He was treated with a cycle of nivolumab and pegylated interleukin-2 on clinical trial and withdrew consent. Standard-of-care ipilimumab and nivolumab were administered between September 2017 and March 2018, with progression in the groin, requiring radiation and continued nivolumab for tumor control. After progression on nivolumab, he was enrolled on an ACT trial (NCT03526185). Tumor was harvested from the right axilla in July 2018 and TIL were administered in August 2018. On first follow-up scans, he had 4% shrinkage (stable disease, confirmed on three subsequent scans), and he remained progression-free until January 2019, when a growing axillary mass was resected, as it was the only site of progression at the time. Re-induction ipilimumab and nivolumab was initiated in May 2019, and in December 2019 he started treatment with binimetinib and encorafenib due to melanoma progression. He succumbed to metastatic melanoma in May 2020.

## Materials and methods

**Fig. 1:**
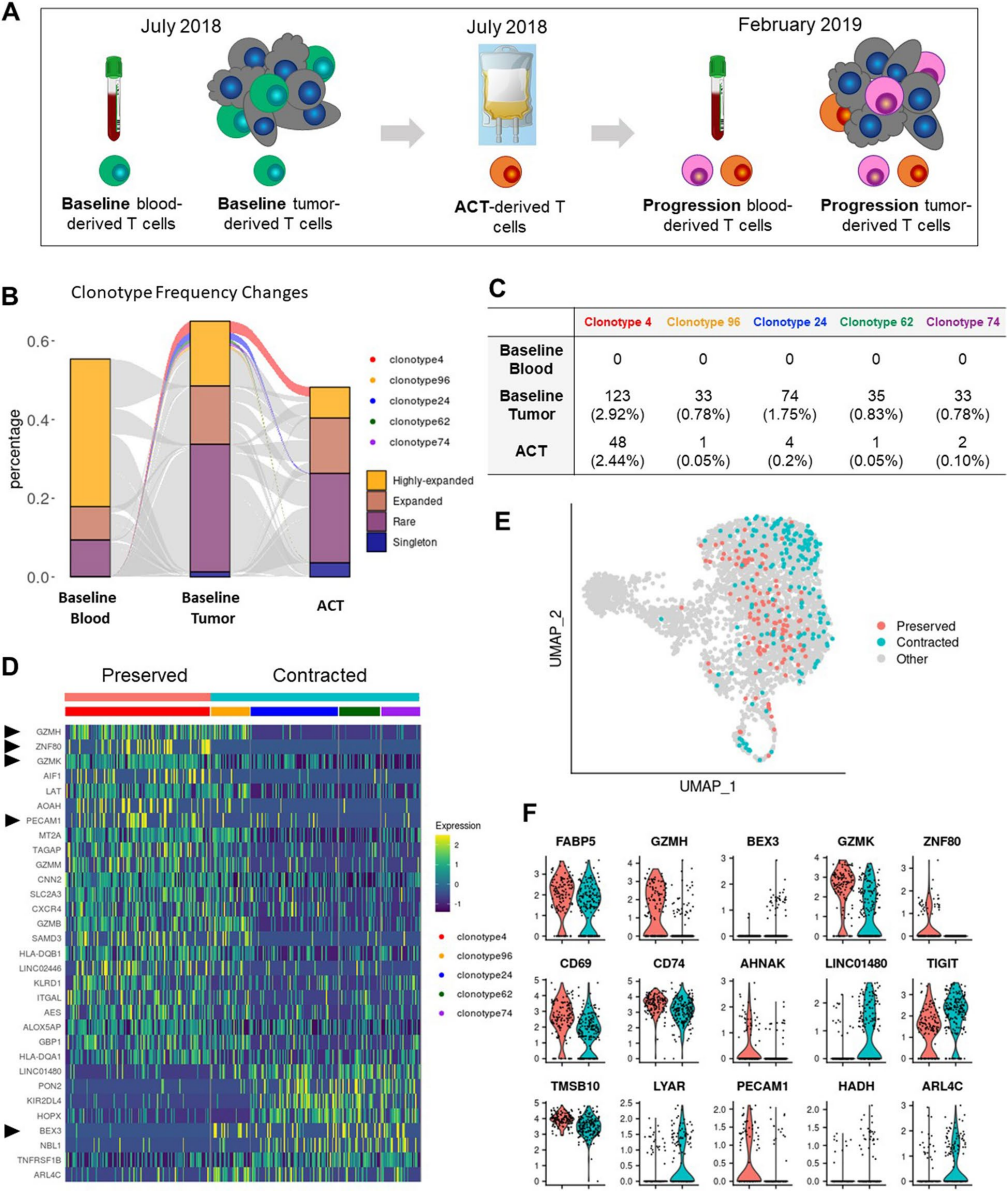
A cytotoxicity signature is associated with clonal persistence during in vitro expansion. **A** Timeline of sample collection. At the recurrence timepoint, orange cells represent T cells derived from the ACT product (based on TcR matching), while pink cells represent endogenous T cells. Bloods at baseline and recurrence were drawn at the time of tumor harvest. **B** Alluvial plot displaying the frequency in onset blood, onset tumor and ACT product of all clonal groups found in at least two of the three samples. Thresholds of expansions are as follows: *singleton* = only one cell in the clonal group; *rare* = the clonal group includes more than 1 cell and occupies less than 0.25% of the sample-specific repertoire; *expanded* = the clonal group occupies between 0.25% and 0.75% of the sample-specific repertoire, *highly expanded* = the clonal group occupies more than 0.75% of the sample-specific repertoire. Clonal groups that are highly expanded in the onset tumor and remain highly expanded in culture are classified as *preserved*. Clonal groups that are highly expanded in the onset tumor and became rare, singletons ore were not found in culture are classified as *contracted*. For ease of comparison, supplemental Fig. 1 includes the same panel without the middle column. **C** Table listing the absolute number of cells with the indicated clonotype, as well as the percentage of the repertoire occupied by each clonal group (in parentheses). **D** Heatmap of differentially expressed genes between expanded TILs belonging to a clonal group that was preserved vs contracted during the ACT culture. Rows represent genes ordered by fold changes. Columns represent individual cells grouped by identity class as indicated on the horizontal bars. Values are scaled and mean-centered log2-transformed gene counts. Arrowheads indicate genes that were also returned by the analysis in F. **E** Scatter plot of UMAP embeddings of onset TILs, colored by belonging to a clonal group that was preserved or contracted in culture. **F** Violin plots of genes identify by feature selection using stochastic gates as discriminating between the region occupied by the cells belonging to the preserved vs. contracted clonal groups

With approval from the Yale University Institutional Review Board, using methods previously described, [2] single-cell RNA-sequencing (scRNA-seq) and TCR-sequencing (TCR-seq) were conducted on TILs from the tumor harvested for ACT (baseline), circulating peripheral blood mononuclear cells (PBMCs) obtained the same day, the infused ACT product, TILs from resection of the progressing tumor removed in January 2019, and PBMCs obtained concurrently (Fig. 1A and supplemental materials). Clonotypes were identified by TCR-seq (supplemental materials), and transcriptomic analyses were conducted (supplemental materials.)

## Results, discussion and conclusions

As shown in Fig. 1B, several T-cell clones from the baseline tumor were identified in the baseline PBMCs. We focused the analysis on five clones that were expanded (> 0.75% of the repertoire) in the initial tumor, clonotypes 4, 24, 62, 74 and 96. Notably, the frequency of the most expanded clone was only 2.92%. These clones were selected because they displayed opposite behaviors in culture, either remaining expanded (clone 4) or becoming barely detectable (96, 24, 62, 74; Fig. 1C). The preserved clonotype was not dramatically different from the contracting clones (Fig. 1D-E), but had higher expression of granzyme family genes and ZNF80, as identified by differential gene expression analysis and verified by feature selection using stochastic gates to discriminate between groups of clones (Fig. 1D, F).

**Fig. 2:**
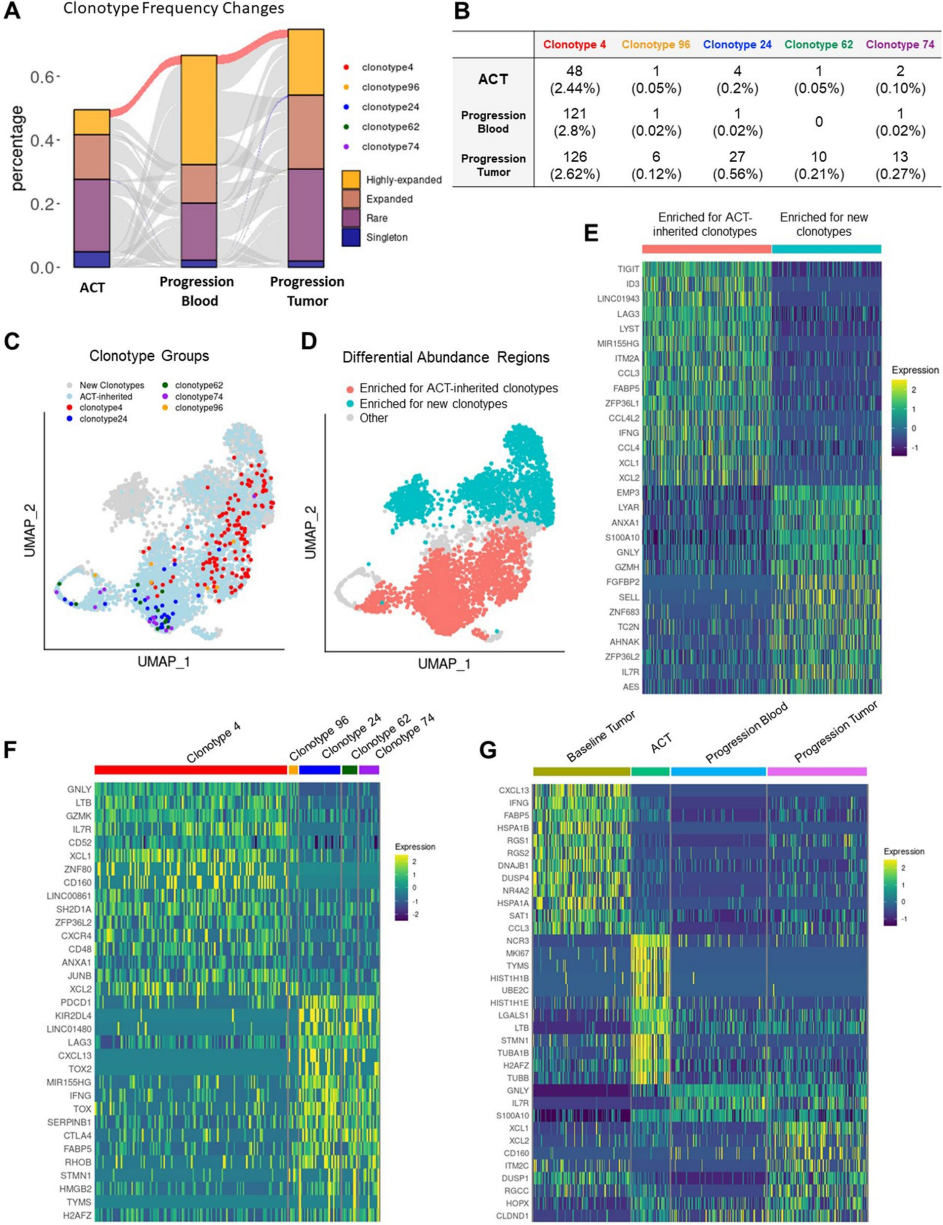
ACT-derived T cells infiltrate a recurring tumor but become exhausted. **A** Alluvial plot displaying the frequency in the ACT blood, tumor at progression and blood at progression of all clonal groups found in at least two of the three samples. Previously defined clonal groups that were preserved or contracted during the ACT culture are highlighted. For ease of comparison, supplemental Fig. 1 includes the same panel without the middle column. **B** Table listing the absolute number of cells with the indicated clonotype, and the percentage of the repertoire occupied by each clonal group (in parentheses). **C** Scatter plot of UMAP embeddings of TILs at progression, colored by expression of the indicated clonotypes. **D** Scatter plot of UMAP embeddings of TILs at progression, showing regions with differential abundance detected between ACT-inherited clonotypes and new clonotypes. **E** Heatmap of differentially expressed genes between cells belonging to the indicated differential abundance areas. **F **Heatmap of differentially expressed genes between cells belonging to the indicated clonotypes in the tumor at progression. **G** Heatmap of differentially expressed genes between cells belonging to clonotype 4 harvested at the indicated time points. For all heatmaps, rows represent genes ordered by fold changes. Columns represent individual cells grouped by identity class as indicated on the horizontal bars. Values are scaled and mean-centered log2-transformed gene counts

Similar analyses were conducted to compare clones in the ACT product to clones at progression and to analyze new clones in blood and tumor at progression (Fig. 2A). Clone 4 remained expanded in both blood and tumor at progression, whereas clones 24, 62, 74 and 96, which had contracted in the ACT product, re-emerged in the tumor at progression, but not in the blood (Fig. 2B-C), indicating that these exhausted cells were incapable of expansion by standard methods, but could still be maintained in vivo. T-cells seen for the first time in the ACT product differed from the new tumor clonotypes at progression (Fig. 2D). The new clones had a more cytotoxic and less exhausted gene signature than sister clones of the ACT product (Fig. 2E). Clone 4 persisted in the tumor at progression (Fig. 2F), however its gene expression profile evolved (Fig. 2G). At baseline, clone 4 cells displayed a terminally differentiated phenotype. In culture, as they proliferated, they upregulated *MKI67, STMN1*. At recurrence, clone 4 cells in the blood had some functional reserve (high in *IL7R*), while in the tumor they expressed markers of precursors of exhausted TILs (*XCL1* and *XCL2*), but without a full-blown exhausted profile [3]. A database search (TCRdb http://bioinfo.life.hust.edu.cn/TCRdb/#/ and VDJbd https://vdjdb.cdr3.net/) for TCR amino acid sequences of the five clones under study retrieved an exact match for both the alpha and beta CDR3 only of clone 4, a dual TCR clone. Both the CDR3 beta (CASTLGTGGHEQYF), and one of the two alpha chains (CAVNSGYSTLTF) were previously found in studies of the TcR repertoire of cytomegalovirus (CMV)-infected individuals. We found no matches for the second alpha chain (CLVGSLYNQGGKLIF). Based on previous validations of this approach, [4] we interpret this as strong evidence that clone 4 is in fact CMV-specific.

These analyses, which involve uncommon and highly valuable serial tumor samples, are informative. First, clones in the harvested tumor that expand and remain expanded during ACT manufacturing may not recognize tumor antigen. The likely CMV-specific clone had high levels of granzyme family members (*GSMH, GZMK, GZMB* and *GZM*) compared to clones that contracted during manufacturing, whereas the other clones did not expand and persist. The contracting clonotypes had lower levels of genes associated with T-cell activation and effector functions, such as *HLA-DQB1, LAT, HLA-DQA1,* and *KLRD1 *[5]. Importantly, *CXCR4*, a key receptor for T-cell trafficking to lymphoid follicles, including extra-nodal lymphoid tissue, was relatively under-expressed in contracting clones. CXCR4 in T-cells might be important for homing of cells from the ACT product to the tumor. Conversely, genes with higher expression in contracting clones include the apoptosis-associated gene *BEX3* and genes found in exhausted cells in other scRNAseq studies, such as *LINCO1480*, *TNFRSF1B* and *ARL4C *[2, 3, 6, 7]. Contraction of expanded clones during ACT culture has similarly been reported by Poschke et al. [8] Additional studies are needed to determine whether exhausted T-cells can be rescued from clonal contraction during ACT preparation, while expanding clones should be studied for tumor cell reactivity. Interestingly, despite the over-expression of genes associated with apoptosis in TILs from baseline tumors, clonotypes 96, 24, 62 and 74 were found again in the tumor at progression.

We and others previously showed that subsets of tumor-based clones are found in the blood [2]. However, most clonal groups expanded in the tumor were absent in the blood, indicating that harnessing T-cell clones from the circulation for cell therapy might be challenging. Of note, new clonotypes in the manufactured ACT product that were not abundant in the harvested tumor, were similarly associated with an exhaustion signature. These newer clones had high expression of *PDCD1, TIGIT, LAG3, CTLA4,* and *HAVCR2,* suggesting that although they were not effective in the adoptive cell therapy setting [9], they might be activated with immune checkpoint inhibitors, and clinical trials such as NCT03108495, are using drugs such as pembrolizumab after ACT.

Although the patient’s disease remained stable for a few months, this is likely due to chemotherapy prior to TIL therapy rather than activity of the TIL product, which should be more protracted. Interestingly, at disease progression, all previously-defined clonotypes were detected in the resected tumor, all were exhausted. Clone 4 remained expanded in both blood and tumor at the later time-point. Future trials are warranted that incorporate TCR analysis to determine tumor-specificity of clones in the TIL product. Moreover, at disease progression, other clonotypes were identified that were either new or sparse in the TIL product, and although these also had high expression of genes associated with a cytotoxic phenotype (*FGFBP2, GNLY, GZMH, GZMK*) and markers of stemness such as *IL7R*, *SELL,* and *KLF2,* these too were unable to reject the growing tumor [2, 7, 10]. Further work is needed to determine whether these are tumor-specific, and if so, develop alternative methods for expansion and modulation. At progression, most new clones in the blood similarly expressed markers of cytotoxicity (*GZMH, GZMB, KLRD1, FGFBP2, PRF1, CX3CR1,* and *NKG7*), and these too might be modified and expanded if they target tumor antigens.

In conclusion, our longitudinal analysis of sc-RNA-seq and T-cell clonality in a melanoma patient who did not respond to TIL therapy demonstrates variability in expansion, persistence, and activation of clones. Clones that tended to contract were more likely to exhibit an exhausted phenotype, while the only clone that remained highly expanded and exhibited markers of cytotoxicity was likely CMV-reactive. Clones appearing in the ACT product that were not abundant in the baseline tumor might be activated pharmacologically with inhibitors of PD-1, CTLA-4, TIGIT or LAG-3. At progression, new clonotypes were identified in both blood and tumor, which expressed effector markers, and, if indeed tumor-specific, these might be harnessed for alternative interventions. Longitudinal studies of additional patients receiving TIL therapy are warranted to determine clonotype activity or exhaustion and tumor-specificity in the setting of response or rapid progression after therapy. TIL products should be assayed for expansion of non-tumor-specific clones to avoid proceeding with TIL therapy when likely futile with the goal of minimizing physical and financial toxicity.

## Supplementary Information

**Additional file 1.** **Supplemental methods.**

## Data Availability

Materials are provided in the supplemental information and raw data are available upon request to the corresponding author.

## Abbreviations

ACT: Adoptive cell therapy
TIL: Tumor infiltrating lymphocytes
TCR: T cell receptor
scRNA-seq: RNA-sequencing
TCR-seq: TCR-sequencing
PBMC: Peripheral blood mononuclear cells
CMV: Cytomegalovirus
