# Supplementary material for: Longitudinal single-cell analysis of a patient receiving adoptive cell therapy reveals potential mechanisms of treatment failure
Source: Mol Cancer. 2022 Dec 14;21:219. doi: 10.1186/s12943-022-01688-5 (PMC9749221; doi:10.1186/s12943-022-01688-5)
Supplement: Supplementary file 1 — Additional file 1. Supplemental methods. [file 12943_2022_1688_MOESM1_ESM.docx]

**Supplemental Methods**

***Patient samples and cell isolation***

At initial resection and at recurrence, freshly resected tumor tissue from an inguinal lymph node was mechanically dissociated and digested enzymatically as previously described^1^. The resulting single cell suspension was then used to isolate mononuclear cells through Lymphoprep gradient centrifugation. Mononuclear cells from blood samples were processed similarly. Samples were sorted on a BD FACS Aria II after staining with a viability dye and antibodies for CD45, TCRαβ, CD4, CD8, CD25, CD127 (all from eBioscience). From blood and tumor, we sorted total CD8 (Live/CD45+/TcRαβ+/CD8+), CD4 T effector (Live/CD45+/TcRαβ+/CD4+/CD25^lo/int^), CD4 Treg (Live/CD45+/TcRαβ+/CD4+/CD25^hi^/CD127^lo^) as individual samples. The ACT product and the initial tumor and blood samples were kindly provided by the Advanced Cell Therapy Laboratory in the Department of Laboratory Medicine (Yale School of Medicine) as cryopreserved aliquots. Those were thawed following the protocol CG00039 from 10X Genomics (<https://support.10xgenomics.com/>) and stained and sorted like the recurrence sample. From the initial tumor and blood, we sorted a mixture of CD8 (Live/CD45+/TcRαβ+/CD8+, 85% of sample) and Tregs (Live/CD45+/TcRαβ+/CD4+/CD25^hi^/CD127^lo^, 15% of sample). From the ACT product, a mix of CD8 (Live/CD45+/TcRαβ+/CD8+, 50% of sample) and total CD4 (Live/CD45+/TcRαβ+/CD4+, 50% of sample).

***Single cell RNA library preparation and sequencing***

Libraries were prepared by the Yale Center for Genome Analysis using Chromium Next GEM Single Cell 5' Library and Gel Bead Kit V1 (1000263), Chromium Single Cell Human TCR Amplification Kit (1000252), Dual Index Kit TT Set A (1000215), and Chromium Next GEM Chip K Single Cell Kit (1000286) according to the manufacturer’s instructions. Following quality control (QC) assessment, libraries were subjected to Novaseq 6000 (Illumina) sequencing with 30,000 read pairs per cell for the Single Cell 5' Library and 10,000 read pairs per cell for the TCR library.

**Single cell data pre-processing and QC**

We collected paired 10x scRNAseq and scTCRseq data from the same patient across different time points. For each scRNAseq sample, raw 10X sequencing data was processed into a UMI count matrix by aligning the reads to the GRCh38 genome following the standard 10X Cell Ranger pipeline (v3.0.1). The downstream data analysis was performed using Seurat v3 R package ^2 3^. In the quality control (QC) analysis we excluded poor-quality cells with the number of detected genes < 2.5% quantile (empty droplets) or > 97.5% quantile (doublets), and/or with the percentage of mitochondria gene expression > 30%. A dubious cell population (likely caused by contamination) with no expression of any T cell markers were removed. In addition, TCR genes were removed from the GEX libraries to prevent clustering bias caused by the contribution of variable V(D)J transcripts in major variable components. We decided to focus on CD8 cells from each sample, which were sorted *in silico* based on expression of *CD8A* and *CD8B.* The data from the initial sample were also used in a previous study, sample Met-T-11^1^.

**Dimensionality reduction and visualization**

The UMI count matrix for each sample was first normalized and scaled with default settings in Seurat. Then the 2000 top variable genes were identified, which served as the input to principal component analysis (PCA) for dimensionality reduction. We retained 30 leading principal components for further visualization and cell clustering. The UMAP embedding was used to visualize the cells on a two-dimensional space. ^4^

**Clonotype repertoire analysis**

scTCRseq samples were first integrated for the consistency of clonotype annotation with the V(D)J module of the Cell Ranger pipeline, where the index of clonotype IDs are determined by ranking the percentage of cells belonging to each clonotype. Then, the clonotype information was added into corresponding Seurat objects as part of the metadata for downstream analysis. To track the change of clonotype composition across different samples, we stratified clonotypes into four categories based on their frequencies in each sample. Specifically, we adopted the following criteria: a clone with only one cell represented is denoted as a singleton clonotype; a clone represented by more than one cell but < 0.25% cells is denoted as a rare clonotype; a clone represented by 0.25% ~ 0.75% cells is denoted as an expanded clonotype; a clone represented by > 0.75% cells is denoted as a highly expanded clonotype. In our study, we identified one clone (clonotype-4) that is highly expanded in the baseline tumor and remained preserved as a highly expanded clonotype in the TIL product, as well as four clones (clonotype-24, 62, 74, 96) highly expanded in the baseline tumor but contracted in the TIL product. Notably, none of these five clones was not present in the baseline blood.

**Marker gene identification and feature selection**

Marker genes highly expressed in each specific cell population were identified based on differential expression analysis. Employing FindMarker function in the Seurat package, we used the non-parametric Wilcoxon rank sum test to look at genes expressed in at least 10% of cells in a population. Besides, we also used stochastic gate (STG) ^5^ to select the list of genes that can best classify different clonotype groups. We first randomly selected 80% of the data for training, 10% for validation, and 10% for testing. After standardizing each set, we optimized the regularization parameter lambda and learning rate of the STG model with 100 trials of Optuna (a hyper-parameter optimization software) on the validation set^6^. In the end, we selected genes whose gates converge to 1 in the optimized model.

**Differential abundance analysis**

To find the region of cells in the tumor at progression where they are locally more abundantly represented by either the clonotypes inherited from the TIL product or endogenous clonotypes, we applied a recently established statistical method ^2^ to perform differential abundance (DA) analysis.^7^ Cell-cell affinity graph was constructed based on Gaussian kernel in a local-adaptive manner (K = 25). Cells with adjusted p-values below 0.01 were identified as DA cells. Genes significantly differentially expressed between the two DA cell populations were identified based on the same workflow as described above (see *Marker gene identification and feature selection*).

SUPPLEMENTAL FIGURE 1


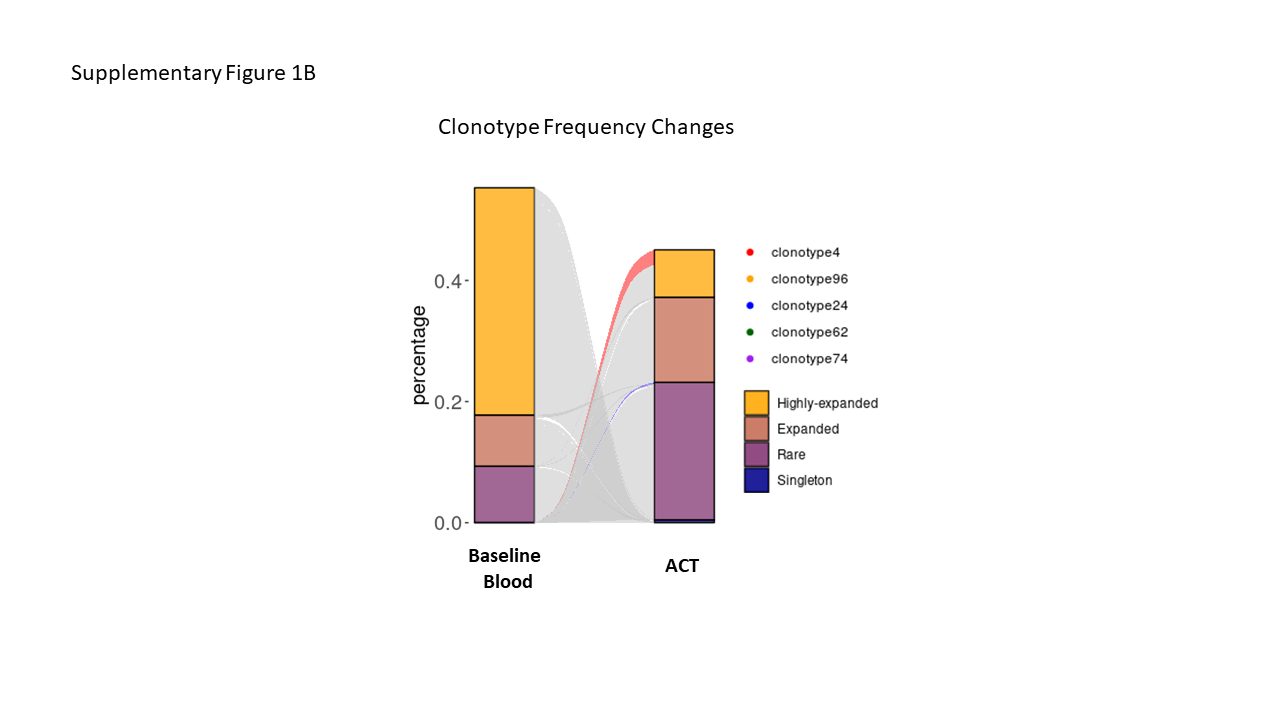


SUPPLEMENTAL FIGURE 2


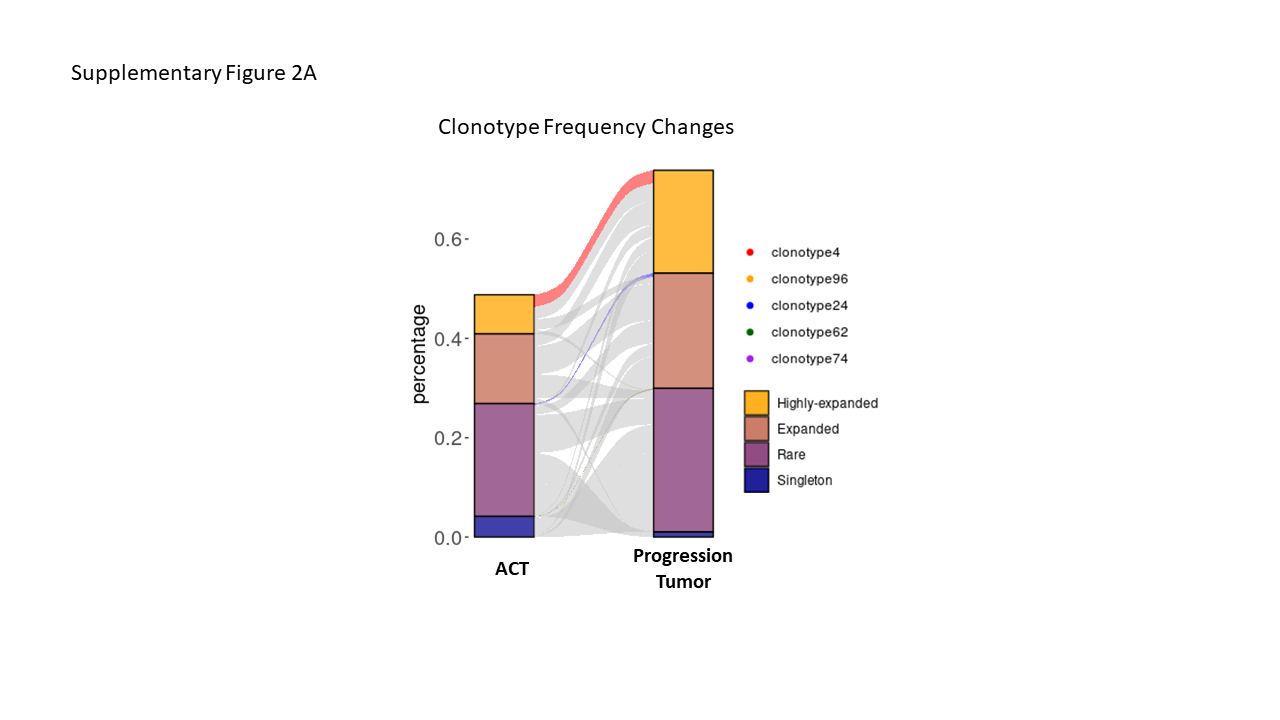


**Supplemental References**

1. Lucca LE, Axisa PP, Lu B, et al. Circulating clonally expanded T cells reflect functions of tumor-infiltrating T cells. J Exp Med 2021;**218**(4).

2. Stuart T, Butler A, Hoffman P, et al. Comprehensive Integration of Single-Cell Data. Cell 2019;**177**(7):1888-902 e21.

3. Satija R, Farrell JA, Gennert D, et al. Spatial reconstruction of single-cell gene expression data. Nat Biotechnol 2015;**33**(5):495-502.

4. Becht E, McInnes L, Healy J, et al. Dimensionality reduction for visualizing single-cell data using UMAP. Nat Biotechnol 2018.

5. Feature selection using stochastic gates. International Conference on Machine Learning; 2020.

6. Akiba T SS, Yanase T, Ohta T, Koyama M. Optuna: A Next-generation Hyperparameter Optimization Framework. arXiv:190710902 2019.

7. Landa B QR, Chang J, Kluger Y. Local Two-Sample Testing over Graphs and Point-Clouds by Random-Walk Distributions. arXiv:201103418 2020.
